# Physical Capacity and Energy Expenditure of Cavers

**DOI:** 10.3389/fphys.2017.01067

**Published:** 2017-12-15

**Authors:** Virginia Pinna, Sara Magnani, Gianmarco Sainas, Giovanna Ghiani, Samuele Vanni, Sergio Olla, Elisabetta Marini, Nicoletta Curreli, Stefano Cabras, Paulo Farinatti, Giorgia Antoni, Filippo Tocco, Andrea C. Rinaldi, Antonio Crisafulli

**Affiliations:** ^1^Department of Medical Sciences and Public Health, University of Cagliari, Cagliari, Italy; ^2^Department of Life and Environmental Sciences, University of Cagliari, Cagliari, Italy; ^3^Department of Biomedical Sciences, University of Cagliari, Cagliari, Italy; ^4^Department of Mathematics and Informatics, University of Cagliari, Cagliari, Italy; ^5^Department of Statistics, Universidad Carlos III de Madrid, Getafe, Spain; ^6^Laboratory of Physical Activity and Health Promotion, University of Rio de Janeiro State, Rio de Janeiro, Brazil; ^7^Gruppo Speleo Archeologico Giovanni Spano, Cagliari, Italy

**Keywords:** heart rate, stroke volume, blood pressure, blood lactate, exercise pressor reflex

## Abstract

Caves are an extreme environment for humans because of the high humidity, mud, darkness, and slippery conditions. Explorations can last many hours or even days, and require extensive climbing and ropework. Very little is known about the physical capacity of cavers and their energy expenditure (EE) during caving. The physical capacity of 17 (7 females) expert cavers (age 43.9 ± 7.3 years) was assessed during an incremental cycle-ergometer test (IET) with gas exchange analysis. Moreover, a wearable metabolic band (Armband Fit Core) was used to estimate their EE during caving. In terms of physical capacity, the IET showed that cavers had a maximum oxygen uptake (VO_2max_) of 2,248.7 ± 657.8 ml·min^−1^ (i.e., 32.4 ± 6.4 ml·kg^−1^·min^−1^), while anaerobic threshold (AT) occurred on average at 74.5% of VO_2max_. Results from caving sessions provided an average time spent in cave of 9.4 ± 1.2 h while the average EE was 268.8 ± 54.8 kcal·h^−1^, which corresponded to about 40% of VO_2max_ measured during IET. A mean distance of 10.6 ± 2.2 km was covered by subjects. Data from the present investigation provide evidence that cavers have a level of aerobic physical capacity only slightly higher than that of sedentary people, thereby suggesting that a high aerobic fitness is not needed by cavers. Moreover, during caving the EE was on average well below the level of AT. However, in absolute terms, the total EE was elevated (i.e., 2,672.3 ± 576 kcal in total) due to the long time spent in caving.

## Introduction

Caves are abundant around the world and are explored for recreational and physical activity purposes by thousands of cavers. Moreover, caves are often explored for scientific purposes. Cave environments are particularly hostile and extreme for humans because of high humidity, mud, darkness, and slippery. They are found in a great variety of settings, from cold alpine to warm tropical rain forests. For this reason, temperature can vary greatly in absolute terms, even though it is usually constant in each cave (White and Culver, 2012). Depths of several hundreds meters and kilometric lengths are not unusual in caves, explorations can last many hours or even days and require extensive climbing and ropework (Gunn, 2004). Thus, given the uniqueness of the environment and the duration of effort, caving can be considered a peculiar sports activity. However, very little is known about the physical capacity of cavers or about their energy expenditure (EE) during caving.

Considering the medical and logistical challenges associated with cave rescue operations, not surprisingly most of the available physiological literature deals with caving injuries (Stella-Watts et al., 2012; Cowart et al., 2014; Schneider et al., 2016). Only some specific studies have addressed the physiological challenge of the Naica Caves, Mexico, where temperature exceeds 45°C and humidity is well over 90% (Giovine et al., 2009) while limited attention has been devoted to the modification of biochemical and hematological parameters during conventional speleological practice (De Felice, 1952; Bregani et al., 2005; Stenner et al., 2006). To the best of our knowledge, to date there is only one study dealing with EE in caving (Antoni et al., 2017), but this investigation is focused mostly on body composition and hydration, while EE in relation to cavers' physical capacity has been only marginally addressed.

Given the scarce information on the EE and level of effort during caving, the physical training of cavers lacks a functional model. Thus, coaches build training programs without any knowledge of the level of effort reached by cavers during explorations.

This investigation was devised to study the physical capacity of a group of skilled cavers. Moreover, the EE during a typical session of a subterranean cave exploration was measured and then compared to the level of physical capacity previously assessed in a laboratory setting to understand the level of effort experienced by cavers in relation to their physical capacity. This information would be useful for coaches to design specific training programs on the basis of the specific EE required by caving.

## Materials and methods

### Subjects

Seventeen (7 females) expert cavers, agreed to take part in the present investigation, which was conducted according to the declaration of Helsinki. They were all Caucasian with at least 5 years of experience in caving. The group mean ± standard deviation (SD) of age, body mass, and height were 43.9 ± 7.3 years, 69.8 ± 15.4 kg, and 167.9 ± 10.9 cm, respectively. Values for women and men were 36.0 ± 8.7 years, 56.4 ± 5.7 kg, 158.2 ± 6.8 cm, and 47.0 ± 5.0 years, 79.4 ± 13.1 kg, 171.9 ± 8.3 cm respectively. Before entering the study, they were carefully screened for their cardiac, pulmonary, and metabolic condition. None of them had any cardiovascular, respiratory or metabolic disease, confirmed by clinical history, basal ECG and physical examination. Written informed consent was signed. The study was approved by the the University Hospital of Cagliari ethics board.

### Experimental design

#### Assessment of physical capacity in the laboratory setting

All subjects underwent an incremental exercise test (IET) on cycle-ergometer (CUSTO Med, Ottobrunn, Germany) to assess their anaerobic threshold (AT), maximum workload (W_max_) and maximum oxygen uptake (VO_2max_). Measurement of gas exchange was performed with a gas analyzer (ULTIMA CPX, MedGraphics St. Paul, MN) calibrated immediately before each IET. The test consisted of a linear increase of workload (20 W/min), starting at 20W, at a pedaling frequency of 60 rpm, until exhaustion, which was taken as the point at which the subject was unable to maintain a pedaling rate of at least 50 rpm. Achievement of VO_2max_ was considered as the attainment of at least 2 of the following criteria: (1) a plateau in VO_2_ despite increasing workload (<80 ml·min^−1^); (2) respiratory exchange ratio (RER) above 1.10; and (3) heart rate (HR) ± 10 beats·min^−1^ of predicted maximum HR calculated as 220-age (Howley et al., 1995). During the IET, AT was determined using the V-slope method, which detects AT by using computerized regression analysis of VO_2_ slopes vs. the carbon dioxide production (VCO_2_) plot during exercise (Beaver et al., 1986). VO_2max_ was calculated as the average VO_2_ during the final 30 s of the incremental test. EE (expressed as kcal·min^−1^) at W_max_ and AT was calculated by utilizing the Weir equation (Mansell and Macdonald, 1990; Ghiani et al., 2015):

$$\begin{array}{l} \text{EE}=3.941•{\text{VO}}_{2}+1.106•{\text{VCO}}_{2} \end{array}$$

This equation was used when the respiratory exchange ratio (RER) was <1, while an oxygen caloric equivalent of 5.04 was used when EE became >1. In this case it was assumed that all aerobic energy derived from carbohydrate oxidation.

All measurements were carried out in a temperature-controlled, air-conditioned room (temperature set at 22°C and relative humidity 50%).

### Measurement of energy expenditure during caving

Energy expenditure during caving was measured by using a wearable device (Armband Fit Core, BodyMedia, Pittsburgh, PA, USA) which has been proven valid for measuring daily energy expenditure under free-living conditions (Johannsen et al., 2010). The Armband is equipped with sensors measuring skin temperature, heat flux, galvanic skin response, and a biaxial accelerometer records as a sensor of movement. The physiological data obtained were then annualized and elaborated by a dedicated algorithm able to provide measures of total EE, total number of steps (TS), and physical activity duration (Drenowatz and Eisenmann, 2011).

Measurements during caving were performed in the Su Palu cave (no. 1988 SA/OG of the Sardinian regional registry of caves, https://www.catastospeleologicoregionale.sardegna.it/). This cave is located in the central-east side of the island of Sardinia (Italy) in coastal area called *Gulf of Orosei*. Su Palu cave is part, together with the Su Spiria-Monte Longos cave, of the Codula Ilune karst system, which is one of the largest in Europe and extends for over 70 km (Arrica et al., 2014). The Su Palu cave has been selected considering its size and the proper combination of the elements which cavers usually face up with during their explorations, i.e., pitches, extensive sub-horizontal galleries, narrow passages, subterranean rivers, and lakes. The internal temperature is around ≈12–15°C, with a tendency to be on the lower side of the range in parts with active waters. Temperature is constant during the year (De Waele, 2004). The underground exploration followed a common route for all cavers. Over a total distance of ≈ 3 km, about 20% has been spent on ropes (both vertical and horizontal), 5% crawling and for sump passages, 15% descending or ascending drops and cliffs without ropes, and the rest walking in sub-horizontal galleries and along rivers. Ground substrate is mostly rocky (not much mud is present). The total difference in height from entrance to arrival point was about 100 m, of which 60 m on ropes. Other morphological details of the cave can be found in its map located at: https://www.catastospeleologicoregionale.sardegna.it/rilievo-pdf/1988. In the internal part of the cave (i.e., not in close proximity to the entrance), air relative humidity is 95–100%, a normal value for caves with active waters (De Waele, 2004).

Cavers entered the cave between May and July 2015. The underground exploration began in the morning (around 10:00 a.m.) and ended in the evening of the same day (between 06:00 and 08:00 p.m.). While caving, all participants moved continuously with the exception of short technical pauses to pass through narrow passages and to rope. Moreover, a brief half-an-hour rest before beginning the way back toward the exit was allowed.

### Statistical analysis

Data are reported as mean ± SD. Descriptive statistics were performed to confirm the assumptions of normality by means of the Kolmogorov-Smirnov test. The alpha level was set at *P* < 0.05. Comparison between EE at AT and at W_max_ of the IET and the average EE during caving was performed by means of the repeated measures analysis of variance (ANOVA), followed by Neuman-Keuls *post-hoc* if appropriate. Significance was set at a *P* < 0.05. Statistics were calculated employing commercially available software (Graph-Pad Prism).

## Results

All participants completed the study protocol. The Kolmogorov-Smirnov test confirmed the normal distribution for all the parameters examined.

**Figure 1:**
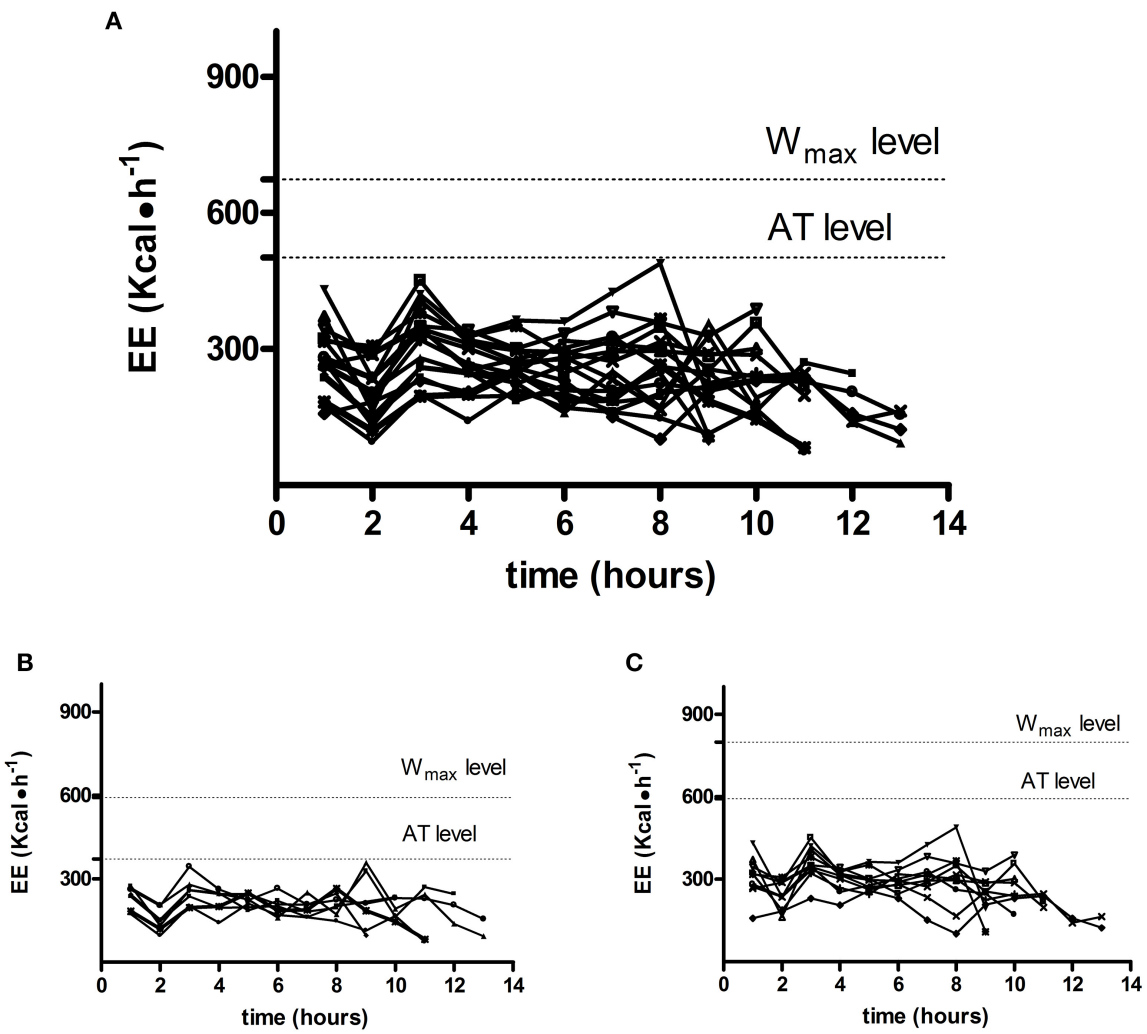
**(A)** Shows the EE time course (1 h average) in each subject during caving. Two horizontal dotted lines identify the EE average group level reached at W_max_ and AT during the preliminary incremental exercise test. **(B,C)** Show separate graphs for female **(B)** and male **(C)** subjects.

**Figure 2:**
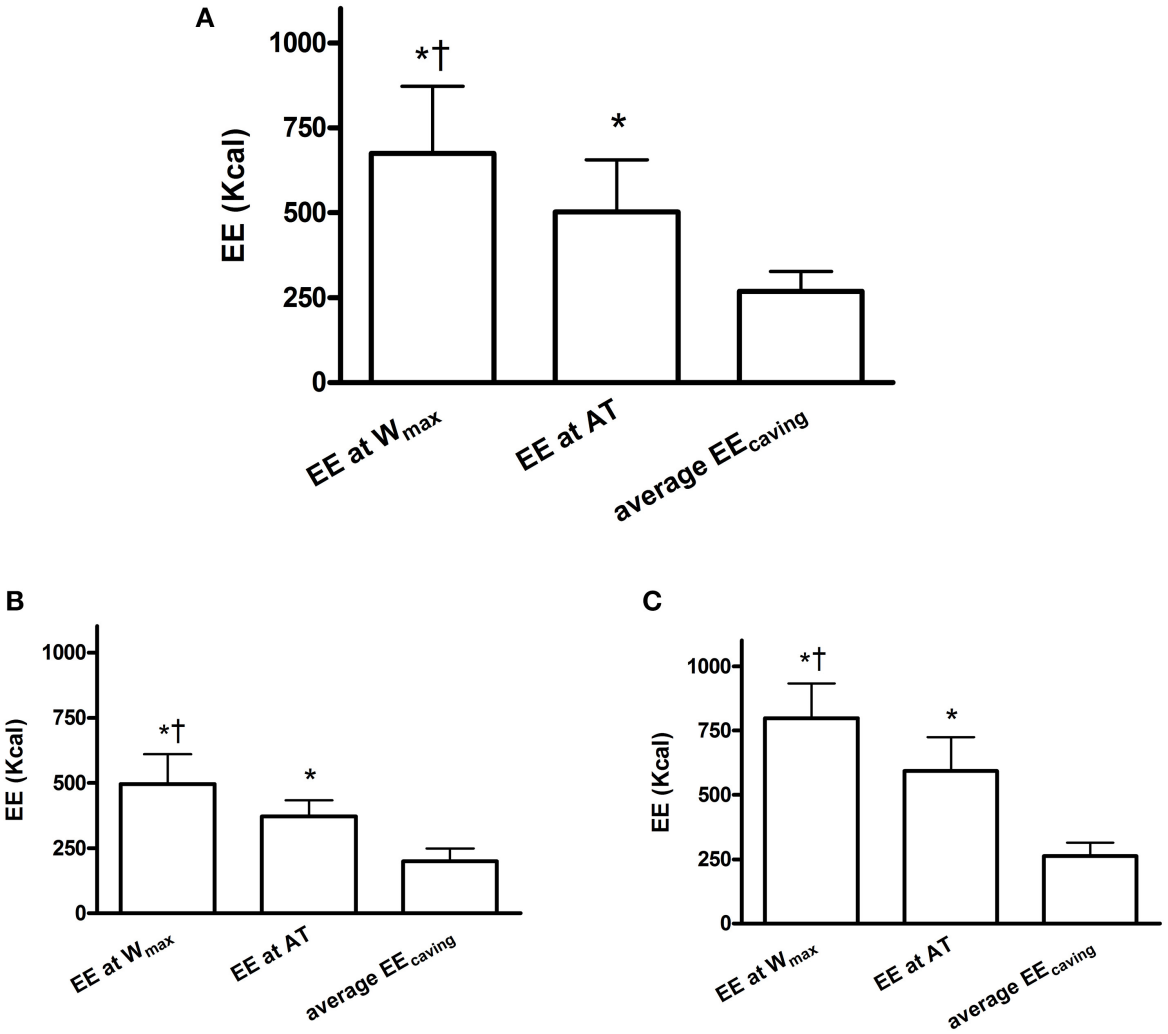
**(A)** Shows results of the ANOVA test applied to the mean values of EE at W_max_ and AT of the preliminary incremental exercise test and the average EE during caving. **(B,C)** Show separate graphs for female **(B)** and male **(C)** subjects. ^*^p < 0.05 vs. average EE_caving_, ^†^*p* < 0.05 vs. EE at AT.

Table 1 shows the results of the IET. In detail, cavers had a maximum oxygen uptake (VO_2max_) of 2,248.7 ± 657.8 ml·min^−1^ (i.e., 32.4 ± 6.4 ml·kg^−1^·min^−1^). The level of W_max_ was 205.7 ± 14.5 W, while anaerobic threshold (AT) occurred on average at 74.5% of VO_2max_. Figure 1A shows the EE time course (1 h average) in each subject during caving. Two horizontal dotted lines identify the EE mean group level reached at W_max_ and AT during the IET. Figure 1 also shows separate graphs for female and male subjects (Figures 1B,C respectively). Results of the ANOVA test applied to the mean values are displayed in Figure 2A, while panel Figures 2B,C shows separate data for female and male subjects respectively. From this figure it can be gleaned that EE at W_max_ was higher than EE achieved at AT (vs. 674.3 ± 197.7 vs. 502.9 ± 154.6 kcal·h^−1^) and also higher than the average EE during caving. Moreover, EE at AT was more elevated than during caving (502.9 ± 154.6 vs. 268.8 ± 54.8 kcal·h^−1^).

**Table 1:** Mean group values ± SD (*n* = 17) of maximum oxygen uptake (VO_2max_, expressed as absolute and indexed by body mass values), maximum carbon dioxide production (VCO_2max_), maximum heart rate (HR_max_), oxygen uptake at anaerobic threshold (VO_2AT_), carbon dioxide production at anaerobic threshold (VCO_2AT_), and heart rate at anaerobic threshold (HR_AT_) reached by subjects during the preliminary incremental test.

	**VO_2max_**	**VO_2max_**	**VCO_2max_**	**HR_max_**	**VO_2AT_**	**VO_2AT_**	**VCO_2AT_**	**HR_AT_**
	**ml·kg^−1^·min^−1^**	**ml·min^−1^**	**ml·min^−1^**	**bpm**	**ml·kg^−1^·min^−1^**	**ml·min^−1^**	**ml·min^−1^**	**bpm**
Mean	32.4	2,248.7	2,888.6	166	24.2	1,676.3	1,868.3	142
*SD*	6.4	657.8	879.6	8.5	5.3	515.2	624.1	8.8

Results from caving sessions provided an average time spent in the cave of 9.4 ± 1.2 h while the average EE was 268.8 ± 54.8 kcal·h^−1^, which corresponded to about 40% of the maximum aerobic capacity measured during the IET. A mean distance of 10.6 ± 2.2 km was covered by subjects. TS were 10,344.8 ± 1,756.4.

## Discussion

The present investigation is aimed at studying the EE in a group of skilled cavers. In particular, we were interested in information on the EE during caving in relation to parameters related to cavers' physical capacity, which was previously assessed with a classical IET. To this end, EE was measured during a typical session of about 10 h subterranean cave exploration. Average EE level was then compared to the level of physical capacity measured in a laboratory setting.

Results suggest that cavers do not need to have a high level of aerobic physical capacity. Actually, the VO_2max_ (i.e., 32.4 ± 6.4 ml·kg^−1^·min^−1^) was only slightly higher than that of a sample (*n* = 15) of sedentary subjects with similar age and body composition selected from the database of our laboratory (30.6 ± 5.8 ml·kg^−1^·min^−1^). This suggests that a high aerobic fitness is not needed to cope with the energy demands of caving. Moreover, throughout caving the EE was on average well below the level of AT. Furthermore, looking at the EE time course of each subject (Figure 1A) it can be gleaned that EE was quite stable in all subjects, without showing abrupt increments and always below the average AT level. Thus, results indicate that caving is not physically demanding. However, in absolute terms, the total EE was high (i.e., 2,672.3 ± 576 kcal in total during a single session) because of the long time spent in exploration. This may be challenging for the body homeostasis, and suggests that the assumption of caloric food portions may be recommendable during long lasting explorations.

Notwithstanding the fact that participants performed well below the level of AT and that their physical fitness was not high in terms of aerobic capacity, it should be taken into consideration that explorations need climbing, rappelling, and ropework (Gunn, 2004). Hence, a good level of muscular strength is probably essential during caving. Moreover, this kind of effort, which can be prolonged for several hours, may increase considerable fatigue, thereby increasing the risk of injuries. It is then conceivable to speculate that specific training programs aiming at increasing muscle strength are useful in this activity.

In the past, several investigations have employed HR in an attempt to estimate EE during various kinds of exercise and daily activities (Gilman and Wells, 1993; Li et al., 1993; Hiilloskorpi et al., 1999; Achten and Jeukendrup, 2003; Green, 2011). The development of accurate and inexpensive telemetry HR monitors has made this method attractive and is widely utilized in freely moving athletes during their field performance. This method is based upon the assumption that there is a direct relationship between HR and VO_2_, which in turn reflects EE. Thus, EE may be calculated from HR. However, while the HR/VO_2_ relationship assessed during incremental laboratory exercise testing is stable and reliable, in the field setting, i.e., when a subject moves freely without a fixed incremental protocol, this relationship is affected by several potentially perturbing factors. In these situations HR may not reflect VO_2_ accurately. One of these conditions is the phenomenon of “cardiac drift,” which appears when exercise duration exceeds 20 min or longer and causes HR to rise slowly and continuously despite stable VO_2_ values. Also heat stress and dehydration increases HR with respect to the real metabolic engagement. All these situations may perturb the HR response, which in turn results excessively high for a given VO_2_, thus overestimating the athlete's actual EE (Crisafulli et al., 2006; Milia et al., 2014; Tocco et al., 2015). Taking into consideration these limits, i.e., the long duration of these sessions, the possibility of heat stress and dehydration during caving, we preferred not to use HR to monitor the EE.

One potential limit of the present study was that *two* methods of measuring of EE were employed: indirect calorimetry conducted in a laboratory setting and the Armband Fit Core during field testing. Although the Armband Fit Core has been proven sufficiently accurate in estimating EE in comparison with indirect calorimetry (Johannsen et al., 2010; Brazeau et al., 2016), it should be noticed that this device has several times been found to underestimate EE during high-intensity exercise (Drenowatz and Eisenmann, 2011; Koehler et al., 2011; Benito et al., 2012; van Hoye et al., 2014). Therefore, we cannot know whether in the present study a systematic underestimation of EE was introduced because of the presence of bouts of high intensity effort during caving. Moreover, considering that climbing and rappelling require the recruitment of anaerobic energy sources, it is reasonable to assume that a substantial amount of energy requirement was derived from anaerobic metabolism. However, the Armband Fit Core has been validated only against indirect calorimetry, which is able to measure EE derived from the aerobic metabolism but not that obtained from anaerobic energy sources. Thus, the Armband Fit Core probably underestimated the total EE for *two* at least *two* reasons: because of its low reliability during high-intensity exercise bouts and because it did not take into consideration the energy derived from the anaerobic energy sources.

This is the *first* study to assess EE during real caving in relation to cavers' physical capacity. Results show that cavers did not need a high aerobic capacity to meet the EE demands of caving in the setting of our experiment However, in absolute terms the total EE was high because of the long duration of sessions. The present information could be useful for coaches to build dedicated training as well as diet programs able to develop the specific adaptations and food intake required by this activity.

## Author contributions

VP, SM, GS, GG, GA, FT, AR, and AC conceived the study, conducted experiments, designed and wrote the paper. SV, SO, EM, NC, SC, and PF conducted experiments, designed and wrote the paper. All authors read and approved the final version of the manuscript.

### Conflict of interest statement

The authors declare that the research was conducted in the absence of any commercial or financial relationships that could be construed as a potential conflict of interest.
